# Relationship between the Formation of Magnetic Clusters and Hexagonal Phase of Gold Matrix in Au_x_Fe_1−x_ Nanophase Thin Films

**DOI:** 10.3390/nano12071176

**Published:** 2022-04-01

**Authors:** Claudiu Locovei, Cristian Radu, Andrei Kuncser, Nicusor Iacob, Gabriel Schinteie, Anda Stanciu, Sorina Iftimie, Victor Kuncser

**Affiliations:** 1 National Institute of Materials Physics, Atomistilor 405A, 077125 Magurele, Romania; claudiu.locovei@infim.ro (C.L.); cristian.radu@infim.ro (C.R.); andrei.kuncser@infim.ro (A.K.); nicusor.iacob@infim.ro (N.I.); schinteie@infim.ro (G.S.); anda.stanciu@infim.ro (A.S.); 2 Faculty of Physics, University of Bucharest, Atomistilor Street 405, 077125 Magurele, Romania; sorina.iftimie@fizica.unibuc.ro

**Keywords:** self-organized magnetic clusters, unidirectional anisotropy, hcp-Au in nanophase Au-Fe thin films, type of magnetic order

## Abstract

Au_x_Fe_1−x_ nanophase thin films of different compositions and thicknesses were prepared by co-deposition magnetron sputtering. Complex morpho-structural and magnetic investigations of the films were performed by X-ray Diffraction, cross-section Transmission Electron Microscopy, Selected Area Electron Diffraction, Magneto Optical Kerr Effect, Superconducting Quantum Interference Device magnetometry and Conversion Electron Mössbauer Spectroscopy. It was proven that depending on the preparation conditions, different configurations of defect α-Fe magnetic clusters, i.e., randomly distributed or auto-assembled in lamellar or filiform configurations, can be formed in the Au matrix. A close relationship between the Fe clustering process and the type of the crystalline structure of the Au matrix was underlined, with the stabilization of a hexagonal phase at a composition close to 70 at. % of Au and at optimal thickness. Due to different types of inter-cluster magnetic interactions and spin anisotropies, different types of magnetic order from 2D Ising type to 3D Heisenberg type, as well as superparamagnetic behavior of non-interacting Fe clusters of similar average size, were evidenced.

## 1. Introduction

In a very short time period, the research field of nanomaterials evolved tremendously due to the novel phenomena and energetically efficient effects that can be harvested from them [1,2,3]. One of the current challenges is to combine the specific features from different types of nanoscale systems to obtain multifunctional nanostructures [4,5,6]. For this purpose, various nanomaterials have been synthesized for their catalytic [7], optoelectronic [8], biomedical [9], and energy storage applications [10]. A system built from at least two non-miscible elements might behave not only as a nanophase structure sharing the common properties of the constituent materials, but may sometimes exhibit new characteristics, not present in the separate materials, which can be revealed by specific clustering effects [11]. For example, if one of the components is of a magnetic nature, such features usually stem from interactions present at interfaces between the involved magnetic nanophases and the embedding matrix. As a result, they can enhance some of the magnetic properties of the system as coercive fields, Curie temperatures and unidirectional anisotropy [12,13], or other related characteristics such as electronic transport, catalytic efficiency, and crystal structure [14]. Moreover, various types of multilayer systems comprised of magnetic/noble metal thin films exhibit the possibility to achieve very good biosensing capabilities through magneto-optical effects [15]. Such effects can be further engineered if the nanostructures are obtained in a nanogranular form [16].

The way that a material responds to an optical, electrical, or magnetic actuation is mostly dictated by its crystal structure that imposes specificities in density of states and the allowed energies of electrons [17]. There are studies reporting how band gap values together with the position of conduction and respective valence band change with the modification of crystal structures of certain semiconductors [18]. Moreover, in the case of metals, the crystal structure bears a tremendous role in the catalytic, electrical, optoelectronic, and magnetic properties [19]. There is a huge interest in studying the electronic properties and associated functionalities in the less common crystalline structure of metals. Some studies reported on the synthesis of metastable structure of hexagonal close-packed nickel (hcp Ni) in the form of nanoparticles or thin films, where resistivity measurements indicated values approximately one order of magnitude higher compared with the usual face-centered cubic (fcc) structure of Ni in systems with similar geometries [20]. Moreover, the saturation magnetization was significantly reduced whereas the catalytic properties were considerably improved with respect to those offcc Ni [21]. Moreover, it is quite challenging to obtain a polymorphism of noble heavy metals or to achieve a stable multifunctional nanomaterial with polymorphic nanostructures, as may be noticed from the paucity of literature on this subject. If magnetic nanoclusters can be formed in such polymorph matrices, a large potential of tuning the magnetic and electronic properties of the nanocomposite system might be achieved. In particular, interesting electron conduction phenomena and magnetic scattering mechanisms can be observed and engineered in gold–iron (Au–Fe) nanosystems, if there is an ability to control both the clustering of the magnetic atoms and the crystalline structure of the Au matrix. The hexagonal close-packed Au was synthesized mainly on a graphene or germanium substrate through wet chemistry methods, and respective molecular beam epitaxy [22,23,24]. Due to the difficulty encountered in the hcp Au synthesis, the electronic properties of this nanomaterial remain not fully analyzed. Furthermore, the behavior of nanostructures comprised of hcp Au (single phase or in combination with fcc Au) and other magnetic nanomaterials represent an unexplored field of research.

In this work, we report on the successful preparation of Au_x_Fe_1−x_ nanophase thin films with different thicknesses and compositional ratios of Au/Fe, respectively. As previously reported [11], for certain deposition parameters the self-organization of Fe clusters into lamellar configurations embedded in the gold matrix can occur at ambient conditions. Here, we present the results obtained on similar systems prepared in a systematic manner by varying deposition parameters in a narrow interval, with an in depth analysis on structural and magnetic properties. Cross-sectional Transmission Electron Microscopy highlighted modifications of morphological features for samples synthesized at different thicknesses and elemental ratios, respectively. Moreover, structural analysis conducted by X-ray Diffraction and Selected Area Electron Diffraction, together with a complete set of magnetic measurements also show a high potential for tuning the crystalline and magnetic features of the Au_x_Fe_1−x_ nanophase systems by carefully choosing the deposition parameters. The experimental data highlighted that there is a close relationship between three observed phenomena, namely uniaxial magnetic anisotropy, self-organization of Fe clusters into lamellar-like configuration, and the crystallization of Au in the hexagonal close-packed structure. Innovative magnetic, electric, and optical features may emerge from such functional nanophase systems. To the best of our knowledge, very few studies have been published on the synthesis of hcp phase of gold at a nanometer scale under special preparation conditions, but none of these reports focused on the magnetic properties of nanogranular systems where both the clustering process and the morphism of the Au matrix were controlled.

## 2. Materials and Methods

The samples were prepared by radio frequency (rf) magnetron sputtering co-deposition from two targets, one of Au and the other of Fe, each 2 inches in diameter and of 99.99 at. % purity (Kurt J. Lesker Ltd., St. Leonards-on-Sea, UK). Prior to each deposition, the main chamber was evacuated at a pressure of 7 × 10^−9^ torr and high purity argon was used to achieve the working pressure of 5.8 × 10^−3^ torr. The Au_x_Fe_1−x_ nanophase thin films were synthesized at room temperature onto n-type <100> silicon (Si) substrates (SIEGERT WAFER GmbH, Aachen, Germany), that firstly were ultrasonically cleaned in ethanol. The elemental composition of samples was varied by applying different working powers on the Au target. The film thickness was controlled from the deposition time (see Table 1). Both elemental compositions and film thicknesses were checked by Energy Dispersive X ray spectroscopy and Total Interferometry Contrast. A buffer layer of Au of approximately 2 nm thickness was deposited in situ onto the Si substrate before each deposition.

Electron Transmission Microscopy (TEM) and Scanning Transmission Electron Microscopy—Dark Field (STEM-DF) images were acquired on a JEM ARM-200F microscope (JEOL Ltd., Akishima, Japan) operating at 200 kV voltage, equipped with a corrector for spherical aberration and an “annular dark field” detector for electrons inelastically scattered by the atoms in the sample. Selected Area Electron diffraction (SAED) patterns were collected with the same microscope (the length of the chamber was 80 cm).

In addition to the SAED analysis, structural characterization was performed in the frame of X-ray diffraction (XRD) using a Bruker D8 Advance diffractometer (Bruker AXS, Karlsruhe, Germany) working with Cu_Kα_ radiation (λ_Kα_ = 0.154 nm). The data were recorded at room temperature (RT) in symmetric geometry at an interval of 2θ = 35–90° with an angular step of 0.04°. For quantitative analysis, an angular step of 0.008° was used around the crystalline plane along the preferential orientation and in each case the data were fitted with Voigt profiles.

Superconducting Quantum Interference Device (SQUID) measurements were acquired on a MPMS 7T system (Quantum Design, San Diego, CA, USA) at a temperature of 270 K and at low temperatures down to 10 K, with the magnetic field orientated parallel to the substrate plane. Zero field cooled-field cooled (ZFC-FC) procedures in a measuring field of 100 Oe were also considered in order to investigate the magnetic relaxation effects. The in plane magnetic anisotropy of rf-magnetron sputtering deposited systems was investigated by vectorial Magneto-Optical Kerr Effect (MOKE) where hysteresis loops were collected at different azimuthal angles between the field and a reference direction in the film plane. The ^57^Fe Conversion Electron Mössbauer Spectroscopy (CEMS) was performed at room temperature by placing the samples in a homemade gas flow (He/CH_4_) proportional counter. The local electronic structure and the specific phase evolution and magnetic configuration of Fe atoms in the Au_x_Fe_1−x_ thin films in function to the deposition parameters were envisaged. The substrate plane was in the front of a ^57^Co(Rh) radioactive source (initial activity of 50 mCi), with the γ photons oriented perpendicular to the films. The NORMOS software was used to obtain the fit of CEM spectra [25]. The calibration was performed using an α-Fe metallic placket at room temperature. Isomer shift (IS) values reported relative to α-Fe at room temperature were given in mm/s, similar to the Quadrupole splitting (QS) values. The hyperfine magnetic fields are given in Tesla (T).

## 3. Results and Discussions

### 3.1. Morpho-Structural Characterization

**Figure 1:**
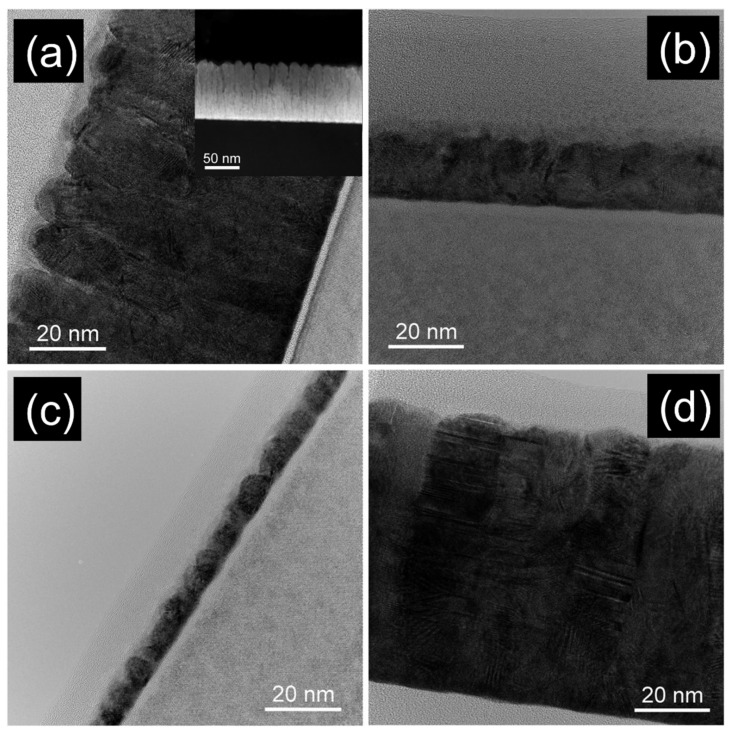
TEM micrographs of sample (**a**) Au_70_Fe_30__70, (**b**) Au_70_Fe_30__17, (**c**) Au_70_Fe_30__6, and (**d**) Au_80_Fe_20__70; the inset from (**a**) contains the STEM-DF image of sample Au_70_Fe_30__70.

The TEM micrographs obtained in the cross-section of the rf-magnetron sputtered thin films are presented in Figure 1. For Au_70_Fe_30__70 sample (70 nm thickness) both the TEM image from Figure 1a and the STEM-DF micrograph from inset suggest the filiform organization of the Fe clusters in the Au matrix. As subsequently proven by magnetic measurements, the real in-depth organization of clusters is of a 2D-lamellar type (with lamella perpendicular to the cross-section STEM-DF image). The co-deposition of Fe and Au induced a growth of gold in a columnar manner separated mainly by Fe clusters self-organized in lamellar configurations (the average size of the Fe clusters is of about 4 nm as also previously reported [11]). The compactness of the layer and the perpendicular orientation of the lamellar 2D organization to the cross section view is evidenced, respectively. The modifications in morphology that occurs in the nanophase thin films when the thickness decreased from 70 nm to 6 nm, while the elemental ratio was maintained constant, are shown in Figure 1a–c. In this set of three samples, a diminishing of the self-assembly effect of Fe clusters was observed for smaller thicknesses, but the layer remained compact without noticeable morphological defects. The variation of the morphological properties of the nanophase system at different compositions was considered as well via Figure 1a,d. By comparing the samples Au_70_Fe_30__70 (Figure 1a) and Au_80_Fe_20__70 (Figure 1d), both of 70 nm thickness, it is seen that the lamellar configuration perpendicular to the substrate plane of the Fe clusters is already becoming suppressed by decreasing the Fe content from 30% at. to 20% at. This behavior was also reported previously for a sample with 15% at. Fe and 85% at. Au, when randomly distributed Fe clusters (also 3–4 nm in size) are formed [11]. As with results of other reports [26,27], when pure Au thin films were prepared at approximately the same deposition parameters without the addition of Fe, compact gold layers with small roughness should be obtained. Noteworthy, the thicknesses of layers from Table 1 can be easily verified by using the scale bar from the TEM micrographs.

**Figure 2:**
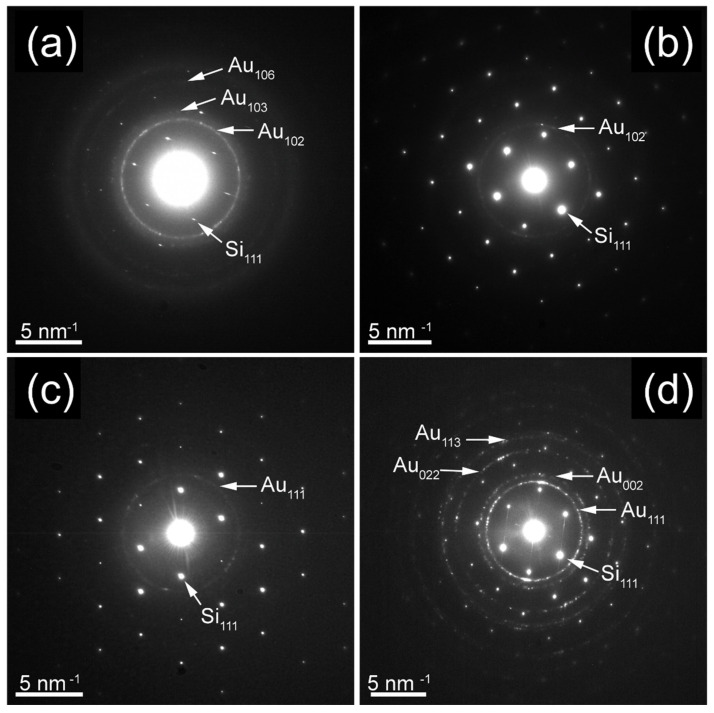
SAED patterns of sample (**a**) Au_70_Fe_30__70, (**b**) Au_70_Fe_30__17, (**c**) Au_70_Fe_30__6, and (**d**) Au_80_Fe_20__70.

The SAED patterns from Figure 2a,b show interplanar distances that match with neither metallic fcc Au nor bcc Fe, respectively. Instead, the values of calculated interplanar distances fit closely for the 4H hcp phase of gold with theoretical lattice constants of a= 2.866 Å and c= 9.662 Å [23]. In this situation, when the composition of the film corresponds to Au_70_Fe_30_, well-defined diffraction rings are observed for the sample of 70 nm thickness and only a faint one in the sample of 17 nm thickness. However, the hcp phase is preserved in Au_70_Fe_30__17 thin film too. In comparison, the SAED patterns of sample Au_70_Fe_30__6 (Figure 2c) present a highly distorted fcc Au, probably since the deposition parameters do not provide, in this situation, the optimum conditions for the Au atoms to condensate into metastable 4H hcp phase. In sample Au_80_Fe_20__70 that contains 80 at. % of Au (Figure 2d) the diffraction rings correspond clearly to only the fcc phase of Au with interplanar distances slightly modified by the presence of Fe.

**Figure 3:**
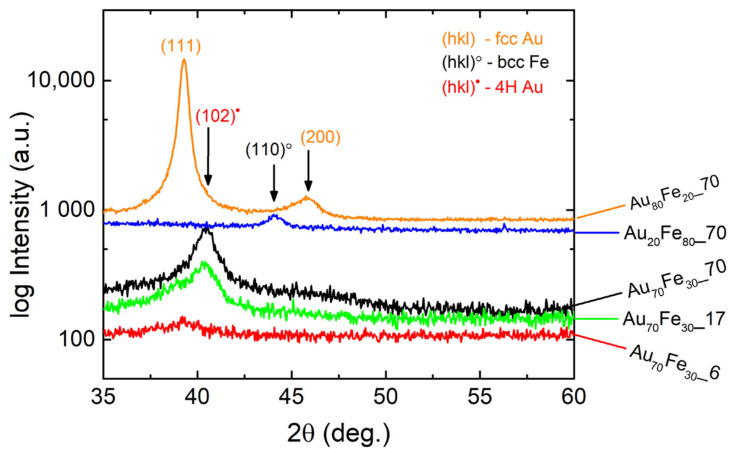
XRD characterization of Au_x_Fe_1−x_ nanophase thin films.

The XRD investigations presented in Figure 3 show the existence of different crystalline phases depending on the composition of samples. Similar with the results from SAED patterns, for Au_80_Fe_20__70 thin films, only the fcc phase of Au with the preferential orientation along the crystalline plane was detected (111). In the case of samples Au_70_Fe_30__70 and Au_70_Fe_30__17, respectively, the diffraction plane (102) of 4H hexagonal Au was mainly observed with a broad signal slightly above background in the case of sample Au_70_Fe_30__70 that may come from the diffraction plane (103), as shown in Figure 2a (traces of the fcc phase are still present). The sample with smallest thickness of 6 nm at the composition Au_70_Fe_30_ shows no defined peak in XRD analysis, but a weak indication for an fcc Au phase at 39° can be observed. Noteworthy, the decrease in thickness from sample Au_70_Fe_30__70 to Au_70_Fe_30__17 leads to a sensible shift in the peak center position and an additional shoulder at 39° (fcc phase), which indicates that for smaller thicknesses, the 4H hexagonal phase of Au becomes less stable. In comparison, the Au_20_Fe_80__70 thin film with the highest amount of Fe presents the body-centered cubic (bcc) phase of Fe where the crystalline plane (110) was highlighted without other crystalline phases.

**Figure 4:**
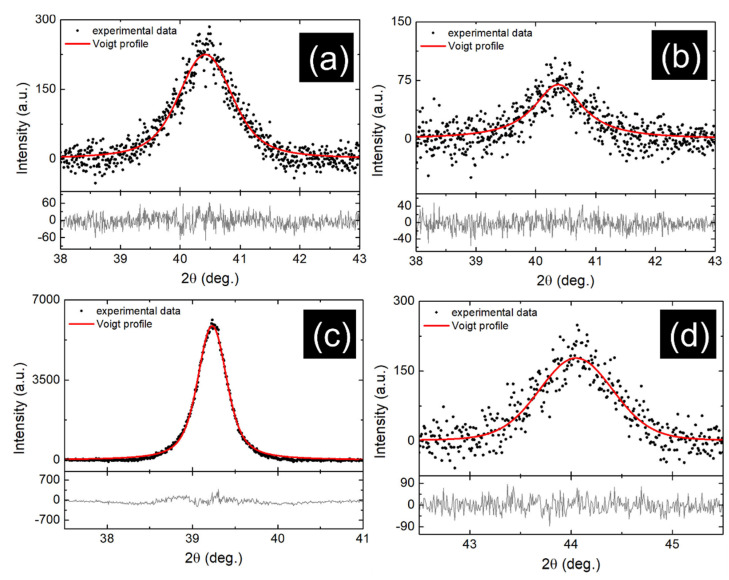
XRD data of 4H Au (102) diffraction peak in sample (**a**) Au_70_Fe_30__70 and (**b**) Au_70_Fe_30__17; fcc Au (111) for sample (**c**) Au_80_Fe_20__70; and bcc Fe (110) in layer (**d**) Au_20_Fe_80__70. The experimental data were fitted with Voigt profiles and the residual of the procedure is present at the bottom of each graph.

In order to determine the structural parameters, XRD measurements were performed in detail around the crystalline planes which gives the crystallographic texture of thin films. Figure 4 shows the experimental data and the theoretical curves obtained by applying Voigt profiles [28]. The residue between experimental and theoretical data are presented at the bottom of each graph. The values of crystallite size ($D_{ef}$) and mean-square micro-stress (${\langle \varepsilon^{2}\rangle }^{1/2}$) specific to the main nanophase are given in Table 2. They were calculated from the integral breadths of the Lorentzian and Gaussian, respectively, of Voigt profile. By comparing the Au_70_Fe_30__70 and Au_70_Fe_30__17 thin films with 4H hcp main phase, it is observed that both $D_{ef}$ and micro-stress decrease with thickness. This indicates a mechanical relaxation between crystallographic planes when the crystalline coherence length is smaller. Moreover, from the values of lattice constants of these two layers it is highlighted that the limits imposed by a lower thickness led to a more deformed unit cell, as was also seen in the SAED patterns. A high value of crystallite size and a smaller micro-stress are specific to sample Au_80_Fe_20__70 where the Au concentration is increased to 80 at. % at the same thickness as for Au_70_Fe_30__70, due to the formation of the more stable fcc Au phase as a main phase. However, in this case the shift in center position of the peak corresponding to the diffraction plane (111) this indicates a structure which is distorted by the presence of Fe. The Au_20_Fe_80__70 film, of much higher Fe concentration evidences mainly the bcc Fe nanophase, with the lattice constant modified by the Au atoms. In addition, in sample Au_20_Fe_80__70 the value of $D_{ef}$ is smaller and micro-stress higher, respectively, than the ones obtained for the Au_80_Fe_20__70 thin film, because Au atoms bear a bigger atomic radius and induce structural defects in the Fe lattice. Furthermore, the surface energy of the (110) crystalline plane of bcc Fe is about 1.5 times higher compared to the surface energy of the plane (111) of fcc Au [29,30]. This would also explain the small size of the Fe clusters in the Au_x_Fe_1−x_ thin films of high Au content as well as the possibility to stabilize mainly the hexagonal Au phase always accompanied by the lamellar structure of the Fe clusters.

### 3.2. Magnetic Investigations

The magnetic texture properties of the films were investigated by vectorial MOKE measurements. Here, possible in plane magnetic anisotropy axes could be highlighted by following the hysteresis loops (Kerr angle versus the magnetic field) collected at different orientations of the applied field with respect to a direction in the sample plane. The idea behind such experiments is that the shape of the hysteresis loop is strongly related to the direction of the field with respect to a possible magnetic easy axis of the system, as theoretically and experimentally proven in [31,32,33]. Accordingly, if the field is oriented along an easy axis (EA) of magnetization, the hysteresis loop will have a quasi-rectangular shape with a jump at a switching field, whereas in the perpendicular direction, the Kerr angle (proportional to the film magnetization) will have a linear variation, reaching the saturation above a saturation field. Such general behavior is still maintained to some extent also in the case of an angular distribution of the easy axis with maximum probability along a certain in plane direction. By counting the wideness of the rectangular hysteresis loop by a so called coercive field (or switching field) or its highness by a so called remanence magnetization, the most rectangular shape of the hysteresis loop can be counted by a maximum coercive field (or remanence) whereas the most linear shape by a minimum (null at the limit) coercive field (or remanence), therefore, the angular dependence of the relative coercive field $H_{C}^{*}=\frac{H_{C}}{H_{C_{\max}}}$ or of the relative remanence magnetization $M_{r}^{*}=\frac{M_{r}}{M_{r_{\max}}}$ versus the angle between the applied field and an in plane reference direction will be very sensitive to magnetic texture effects. Strong bilobar angular distributions will count for uniaxial magnetic anisotropies whereas more spherical angular distributions will count for lack of magnetic anisotropies.

**Figure 5:**
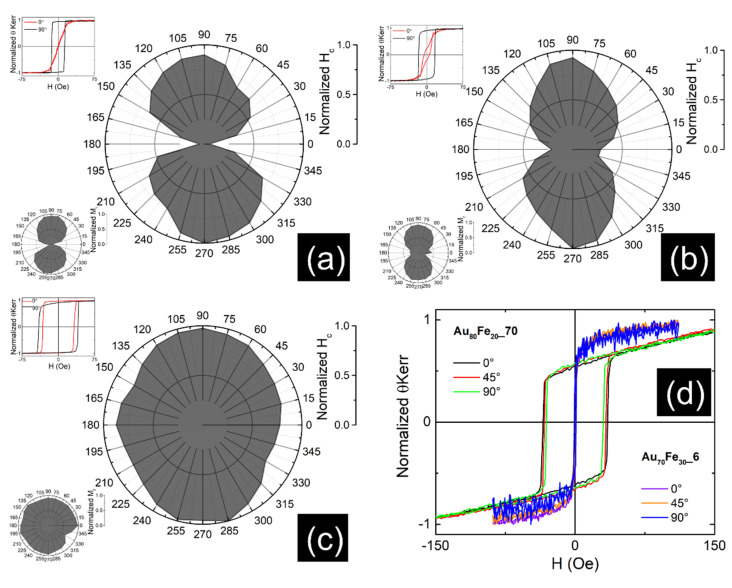
Polar graphical representation of normalized Hc obtained from MOKE hysteresis curves acquired at different azimuthal angles (inset shows the normalized M_r_ in function of recording azimuthal angles and the hysteresis loops corresponding at 0° and 90°) for sample (**a**) Au_70_Fe_30__70, (**b**) Au_70_Fe_30__17 and (**c**) Au_20_Fe_80__70; the hysteresis loops from (**d**) were obtained on sample Au_80_Fe_20__70 and Au_70_Fe_30__6.

The graphical representations of the relative coercive fields ($H_{C}^{*}$) and relative remanence magnetization ($M_{r}^{*}$) in function of azimuthal angles with a step of 15° taken from hysteresis curves recorded on samples Au_70_Fe_30__70 and Au_70_Fe_30__17 are shown in Figure 5a,b, respectively. The magnetic uniaxial anisotropy observed in both samples regardless of the chosen magnetic parameter further supports the formation of the 2D-lamellar organization of the Fe clusters in the Au matrix that was previously observed in TEM micrographs. Because no metallic Fe was detected in SAED patterns or XRD data, the effect of magnetic uniaxial anisotropy can only occur from the self-assembly of the very fine magnetic clusters (of non-observable structural coherence length) along preferential directions parallel to the anisotropy axis. Figure 5b shows a higher dispersion in the dependencies of the two magnetic parameters with the azimuthal angles (less well separated lobs) than Figure 5a. Thus, more localized minima and maxima of normalized H_c_ or M_r_ are highlighted for the Au_70_Fe_30__70 thin film which indicates the better formation of the 2D-lamellar structures of the Fe magnetic clusters in the thicker films with 30 at.% of Fe. In contrast, the Au_20_Fe_80__70 thin film (of higher amount of Fe) presents an almost spherical angular distribution of the $H_{C}^{*}$ and $M_{r}^{*}$ parameters, suggesting that at 80 at. % of Fe, large and percolating magnetic clusters are formed in the film, giving rise to an overall polycrystalline bcc-Fe structure, highly impurified by Au atoms. The hysteresis loops of samples Au_70_Fe_30__6 and Au_80_Fe_20__70 from Figure 5d, whose shapes are insensitive to the rotation angle, show no in plane magnetic anisotropy axis. In the case of both samples, the loops are composed of two components: (i) a fast magnetization reversal at low fields (of almost null coercive field in the case of sample Au_70_Fe_30__6 and with a coercive field of about 40 Oe in case of sample Au_80_Fe_20__70) and (ii) a linearly increasing trend of magnetization in higher magnetic fields of up to hundred Oersted. Nevertheless, the latter component can be assigned to non-interacting magnetic clusters of Fe with superparamagnetic behavior at room temperature whereas the former can be attributed to interacting magnetic clusters, which blocks temperatures depending on the interaction paths and/or cluster size. Such additional peculiarities will be further discussed via SQUID investigations at different temperatures.

**Figure 6:**
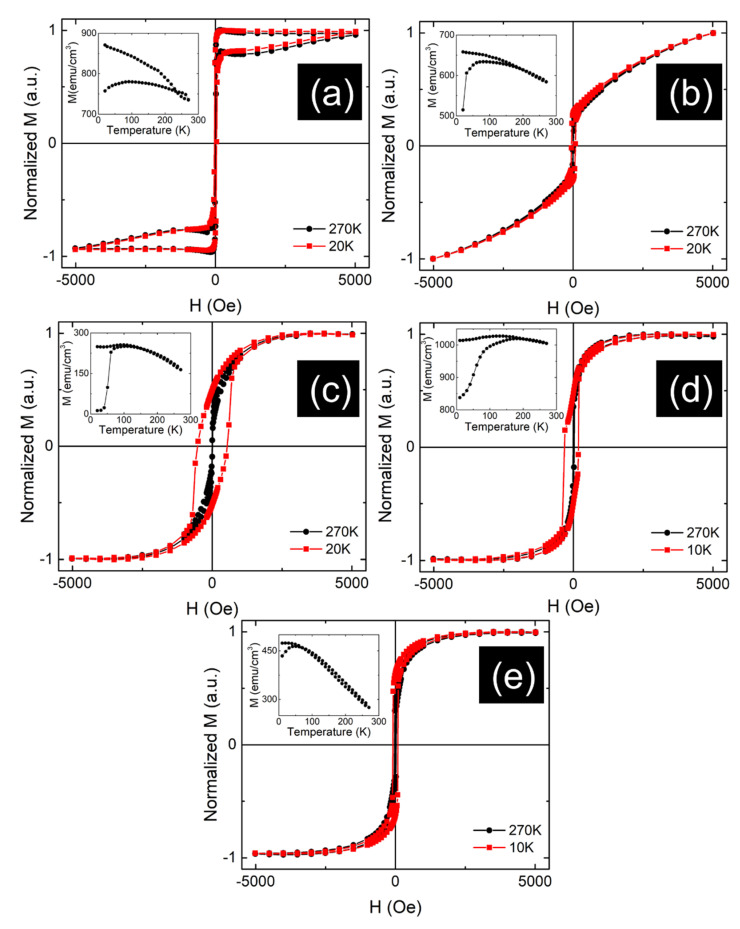
SQUID hysteresis loops of nanophase thin films (**a**) Au_70_Fe_30__70, (**b**) Au_70_Fe_30__17, (**c**) Au_70_Fe_30__6, (**d**) Au_20_Fe_80__70, and (**e**) Au_80_Fe_20__70 (inset presents the ZFC-FC curves for each sample).

Hysteresis loops from Figure 6 were collected by SQUID magnetometry with the magnetic field applied parallel to the substrate/film and along the EA of magnetization (when the system presents one) for two temperatures. The insets show the ZFC-FC curves collected in 100 Oe, for each sample. By observing Figure 6a, a superposition between two magnetic phases is inferred from the shape of the hysteresis loop. This behavior was tentatively assumed as mainly due to the inconsistency of the 2D lamellar organization of the Fe clusters in the sample. Irregular lamellar structures might be formed with a distribution in lengths and inter-lamellar distances down to the formation of separate non-interacting clusters. Therefore, exchange integrals associated with interactions between clusters can drastically change their character, e.g., from 0D (for separate clusters) to 2D magnetic systems (specific to non-interacting lamellar structures of clusters where exchange integrals are finite only within the planar structure of the lamella) and even 3D magnetic systems (specific to the interacting lamellar structure of clusters where exchange integrals are finite along all the three directions of the space), each one imposing different types of magnetic order [34]. The first type of 2D interactions has to respond for the magnetic component of faster magnetic reversal, i.e., completed in tens of Oe, whereas the second type of 3D interactions has to respond for the magnetic component of a much harder magnetization reversal, i.e., completed in thousands of Oe. To note that such interactions are persistent up to room temperature and the MOKE measurements performed in only 75 Oe as presented in Figure 5, provide information only on the ordered magnetic state of clusters organized in non-interacting lamellar configurations which were proven to present uniaxial anisotropy, inferring therefore a 2D Ising type of magnetic system.

The magnetization reversal curves of sample Au_70_Fe_30__17 (see Figure 6b) also provides support for the presence of two magnetic components. The first one of faster magnetization reversal, i.e., completed in tens of Oe, is due to non-interacting lamellar structures of clusters (i.e., a 2D Ising type of magnetic system with uniaxial EA, as in the previous case). The second component of progressive quasi-linear increment of magnetization in higher fields is due to non-interacting magnetic clusters of Fe (i.e., a 0D magnetic system) which are superparamagnetic above 70 K. It is worthy of noting that the amount of such non-interacting Fe clusters, randomly distributed in the Au matrix, is much higher than in the case of the sample Au_70_Fe_30__70. Their massive formation is a direct morphological effect specific to the thinner film, where more fcc Au is formed. At the smallest thickness of 6 nm, the Fe clusters present a superparamagnetic behavior at room temperature and the film thickness impose a rather narrower particle size distribution as reflected in the sharpest reaching of the blocking temperature, also of approximately 70 K (see inset in Figure 6c).

Above 70 K, the ZFC magnetization decreases extremely slowly, showing that a higher number of magnetic clusters are still in a sufficiently high interaction to preserve a certain magnetic order. The involved type of interaction among such clusters can be deduced from the very high coercive field which characterizes them at the low temperature of 20 K. This clearly suggests a high uniaxial anisotropy and presence of magnetic order specific to only a 2D Ising type of magnetic system [34]. This is in agreement with only one layer of Fe magnetic clusters distributed in the film plane, especially along linear chains. Such magnetic chains interact also laterally via the interstitial Fe clusters of similar size, which enter into the magnetic frozen regime below the blocking temperature of 70 K, therefore providing the 2D magnetic character (exchange interactions in the two directions of the film) of the overall system.

Further, the magnetization curves of sample Au_20_Fe_80__70 are shown in Figure 6d. In addition to the soft magnetic character of the much larger magnetically percolating Fe clusters, the presence of a low amount of antiferromagnetic or magnetic disordered phase (probable due to a high amount of Au atoms locally entering the Fe matrix) is suggested by the observed exchange bias field [31] present in the hysteresis loop at low temperatures. Finally, the specific magnetization reversal curves collected at different temperatures for Au_80_Fe_20__70 thin films and the ZFC-FC curves (Figure 6e) showing a weak divergence below a blocking temperature of approximatley 70 K and a similar quasi-linear decrease of the magnetization above 70 K, suggest the formation of randomly distributed Fe clusters with a rather broad sized distribution. Accordingly, excluding the low fraction of the finest Fe clusters which are superparamagnetic above 70 K, the larger ones are in the regime of collective excitations characterized by a linear decrease of the magnetization (or magnetic moment) due to slightly activated incipient magnetic relaxation effects [31].

**Figure 7:**
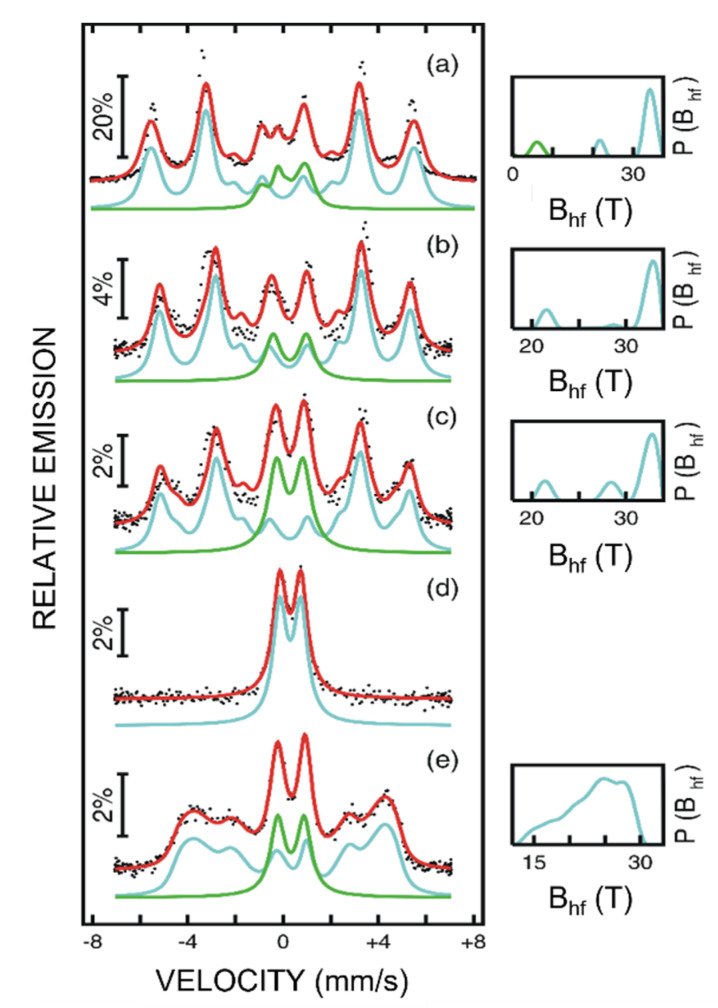
Room temperature Conversion Electron Mössbauer (CEM) spectra of (**a**) Au_20_Fe_80_, (**b**) Au_70_Fe_30_ 70, (**c**) Au_70_Fe_30_ 17, (**d**) Au_70_Fe_30_ 6, and (**e**) Au_80_Fe_20_ 70. On the right side of each spectrum, the corresponding distribution of hyperfine magnetic fields is presented.

The RT CEM spectra of the analyzed Au_x_Fe_1−x_ thin films are shown in Figure 7. The CEM spectrum of the film with the highest Fe content, Au_20_Fe_80,_ presenting the typical structure of bcc metallic Fe and characterized by a very broad magnetic sextet (see Figure 7a) was fitted by two components. A broad external sextet was accounted via a distribution of hyperfine magnetic fields with a main maximum at about 33 T. The corresponding distribution is presented in blue on the right side of the spectrum. A second more central spectral component was accounted by a distribution of hyperfine magnetic fields centered on about 6 T, shown in green in the overall hyperfine magnetic field distribution on the right side of the spectrum. Nevertheless, the hyperfine magnetic field distribution with the main peak at 33 T has to be assigned by its specific hyperfine parameters (most probable hyperfine magnetic field at 33.5(3) T and average IS, of about 0.0 mm/s) to Fe atoms in a defected bcc structure of Fe. Expectedly, this structure is locally distorted due to both the energetic deposition method and the presence of the much larger Au atoms. In fact, under the same distribution a second weak local maximum at about 22 T was observed, being assigned to those Fe positions strongly perturbed by many Au impurity atoms [35]. The local maximum around 6 T of the second distribution is assigned, by the specific value of the average isomer shifts close to −0.14(2) mm/s to large clusters of Fe atoms in the fcc structure of Au, behaving at the limit as a magnetically disordered γ-like Fe phase [35]. This second structural component with a relative spectral contribution of 18(1)% seems to be responsible for the exchange bias field observed in the low temperature hysteresis loop evidenced in Figure 6d. Being magnetically disordered with an antiferromagnetic-like behavior, this magnetic phase pins the soft ferromagnetic phase specific to the main phase of α-Fe, giving rise to specific unidirectional anisotropy effects [31]. The CEM spectrum of Au_70_Fe_30__70 thin film, shown in Figure 7b, was also fitted by two spectral components. The probability distribution of hyperfine magnetic fields resulting from the fitting of the broad external sextet (shown also on the right side of the Mössbauer spectrum) presents a strong maximum at a hyperfine magnetic field of 32.7(2) T and a much weaker one at about 21 T. Nevertheless, by similitude to the hyperfine field distribution of the previously mentioned Au_20_Fe_80__70 thin film, this can also be assigned to Fe atoms in a defected α-Fe phase with or without one or more Au impurities as neighbors of the Fe atoms. Due to the much lower amount of Fe in the Au_70_Fe_30__70 thin film, this external Mössbauer component must correspond to distinct clusters of α-Fe with Au inclusions organized in the lamellar 2D magnetic structures with or without inter-lamellar magnetic interactions. In addition to this magnetic component, a second spectral component accounted by a central broad doublet had to be considered for the most reliable fitting of the CEM spectrum. By its specific average IS value of only 0.15(1) mm/s it can be assigned to the fine and randomly dispersed clusters of α-Fe with Au impurities, which are superparamagnetic at room temperature. Only 18% of the total Fe form the superparamagnetic clusters of random distribution, according to the relative area of the two spectral components in sample Au_70_Fe_30__70. The same two spectral components, an external broad magnetic sextet and a central broad superparamagnetic doublet with IS = 0.16(1) mm/s, were also considered for fitting the CEM spectrum of sample Au_70_Fe_30__17. In the case of this sample, 30% of Fe (almost double that of the thicker film) gives rise to the fine and randomly distributed α-Fe clusters which are superparamagnetic at room temperature, in agreement with the magnetic measurements in Figure 6b. In the case of the thinnest film, Au_70_Fe_30__6, all Fe atoms give rise to clusters of α-Fe. These are superparamagnetic at room temperature despite their Ising type 2D magnetic order evidenced at 20 K via the hysteresis loop shown in Figure 6c. Two additional important traces for the presence of the Au atoms in the bcc structure of the distinct α-Fe clusters in the Au matrix must be mentioned. Both the average quadrupole correction and isomer shift specific to the external sextets were slightly increased in absolute value when the Fe content or the film thickness decreased. For example, the quadrupole shift changes from 0.00 mm/s in Au_20_Fe_80_ film to −0.06(2) mm/s, in the Au_70_Fe_30_ _17 film, whereas the isomer shift changes from 0.00 mm/s to 0.16 mm/s, respectively, as expected by a different number of Fe neighbors around the central Fe in the bcc structure [36]. On the other hand, the intensity ratio R_23_ between the second and the third emission line of the sextet was present in all the above mentioned three samples equal to four, inferring a clear orientation of the Fe spins/magnetic moments in the film plane. Notably, the in plane anisotropy highlighted by the CEM spectra in the cases of the Au_70_Fe_30_ _70 and Au_70_Fe_30_ _17 films, corroborated with the uniaxial anisotropy as pointed by the MOKE investigations provides a final full support for the Ising type of spin anisotropy regarding the lamellar-like organization of the Fe clusters.

Finally, the CEM spectrum of sample Au_80_Fe_20__70 presented in Figure 7e was also fitted with two spectral components, namely a very broad sextet with a relative spectral area of 74 % (IS = 0.3 mm/s) and a central superparamagnetic doublet with a relative spectral area of 26 % (IS = 0.16(1) mm/s). The assignment of the two spectral components is somehow similar to the previous case. The sextet giving rise to the very broad and unshaped hyperfine magnetic field distribution with a local maximum situated at 25.4 T was assigned to highly distorted and larger Fe clusters of a broad sized distribution, strongly unpurified by Au atoms (as sustained by both the relative lower values of the hyperfine magnetic fields and the relatively higher isomer shift values). These clusters are randomly distributed in the fcc-like Au lattice and large enough to carry finite magnetic moments which interact via tridimensional exchange interactions (presenting therefore a 3D magnetic order) even at room temperature, as sustained by the MOKE loop in Figure 5d. In addition, the intensity ratio R_23_ for this sample is close to one, also showing a tridimensional angular distribution of the Fe spins in the film and hence, a Heisenberg type of spin anisotropy. The central doublet was assigned by its specific hyperfine parameters to much finer Fe clusters impurified by Au atoms, which were superparamagnetic at room temperature.

## 4. Conclusions

Au_x_Fe_1−x_ nanophase thin films with compositions ranging from x = 20 to x = 80 at. % and thicknesses ranging from 6 to 70 nm were obtained by co-deposition magnetron sputtering and further investigated with respect to their morpho-structural and magnetic properties. A high potential for tuning the crystalline and magnetic features of the films with respect to both the clustering process of the Fe atoms and the type of crystalline structure of the Au matrix was evidenced. It was proven that at a specific Fe content close to 30 at.%, clusters of α-Fe with specific lamellar organization are formed, mainly in the metastable hcp 4H Au matrix. However, a fraction of randomly distributed Fe clusters with superparamagnetic behavior at room temperature is always formed. The inter-lamellar magnetic interactions can be modified via the film thickness. A shift from a 3D Ising type magnetic order to a 2D Ising type magnetic order was obtained by decreasing the film thickness from 70 nm to 17 nm. For the thinnest film of 6 nm, a filiform organization of the Fe clusters was obtained with induced transversal interaction mediated by randomly distributed Fe clusters in their magnetic blocked regime. The average size of the Fe clusters is close to 4 nm, as proven by both the Transmission Electron Microscopy investigations and the similar blocking temperature of the randomly distributed clusters. The fcc structure of the Au matrix was formed by decreasing the Fe content at 20 at. %. This time, randomly distributed Fe clusters with a much wider size distribution and larger average size were formed in a higher amount. They have a specific lower magnetic relaxation, e.g., in the regime of collective excitation at room temperature. This system was characterized by a 3D Heisenberg type magnetic order.

## Figures and Tables

**Table 1:** Deposition parameters of the RF magnetron sputtering nanophase systems.

Sample *	Deposition Power on Fe Target (W)	Deposition Power on Au Target (W)	Thickness (nm)
Au_70_Fe_30__70	100	25	70
Au_70_Fe_30__17		25	17
Au_70_Fe_30__6		25	6
Au_20_Fe_80__70		10	70
Au_80_Fe_20__70		40	70

* Sample denomination contain the atomic elemental content in % (1 % error) as subscripts and the film thickness in nm as suffix (1–2 nm error).

**Table 2:** Structural parameters of Au_x_Fe_1−x_ thin films calculated from Voigt profile.

Sample	$D_{ef}\;(\mathrm{nm})$	${\langle \varepsilon^{2}\rangle }^{1/2}$	a (Å)	c (Å)
Au_70_Fe_30__70^(102)^	17	7.0 × 10^−3^	2.890	9.823
Au_70_Fe_30__17^(102)^	9	1.3 × 10^−3^	2.894	9.816
Au_80_Fe_20__70^(111)^	39	2.6 × 10^−3^	3.974	-
Au_20_Fe_80__70^(110)^	17	4.6 × 10^−3^	2.905	-
